# Effectiveness of biosimilar pegfilgrastim in patients with multiple myeloma after high-dose melphalan and autologous stem cell transplantation

**DOI:** 10.1007/s00277-023-05228-z

**Published:** 2023-04-20

**Authors:** Massimo Martino, Mercedes Gori, Gaetana Porto, Maria Pellicano, Ludovica Santoro, Chiara Verduci, Filippo Antonio Canale, Barbara Loteta, Tiziana Moscato, Caterina Alati, Maria Consuelo Ieracitano, Amelia Cuzzocrea, Maria Altomonte, Maria Teresa Florenzano, Antonella Morabito, Giuseppe Irrera, Virginia Naso, Marta Pugliese, Giuseppe Console, Anna Ferreri, Lucrezia Imbalzano, Giovanni Tripepi, Annalisa Pitino

**Affiliations:** 1 Stem Cell Transplantation and Cellular Therapies Unit (CTMO), Department of Hemato-Oncology and Radiotherapy, Grande Ospedale Metropolitano “Bianchi-Melacrino-Morelli,”, 89124 Reggio Calabria, Italy; 2 Stem Cell Transplant Program CIC587, 89124 Reggio Calabria, Italy; 3 Institute of Clinical Physiology (IFC-CNR), Section of Rome, 00185 Rome, Italy; 4 Hematology Unit, Department of Hemato-Oncology and Radiotherapy, Grande Ospedale Metropolitano “Bianchi-Melacrino-Morelli,”, 89124 Reggio Calabria, Italy; 5 Pharmacy Unit, Department of Hemato-Oncology and Radiotherapy, Grande Ospedale Metropolitano “Bianchi-Melacrino-Morelli,”, 89124 Reggio Calabria, Italy; 6 Institute of Clinical Physiology (IFC-CNR), Section of Reggio Calabria, 89124 Reggio Calabria, Italy

**Keywords:** Autologous stem cell transplant, Biosimilars, Engraftment, Granulocyte colony-stimulating factor, Multiple myeloma, Pegfilgrastim

## Abstract

Multiple myeloma (MM) is the main indication for autologous stem cell transplantation (ASCT). Novel supportive therapies (e.g., granulocyte colony-stimulating factor) have significantly improved post-ASCT-related mortality; however, data on biosimilar pegfilgrastim-bmez (BIO/PEG) in this setting is lacking. This prospective cohort study compared Italian patients with MM who received BIO/PEG post-ASCT with data collected retrospectively from historical control groups from the same center who received either filgrastim-sndz (BIO/G-CSF) or pegfilgrastim (PEG; originator). The primary endpoint was time to neutrophil engraftment (three consecutive days with an absolute neutrophil count ≥ 0.5 × 10^9^/L). Secondary endpoints included incidence and duration of febrile neutropenia (FN). Of the 231 patients included, 73 were treated with PEG, 102 with BIO/G-CSF, and 56 with BIO/PEG. Median age was 60 years and 57.1% were male. Neutrophil engraftment was reached after a median of 10 days in the BIO/PEG and PEG groups and 11 days in the BIO/G-CSF group. Among patients who achieved neutrophil engraftment earlier than this (i.e., day 9), 58% (29/50) were on PEG; of those who achieved it later (i.e., day 11), 80.8% (59/73) were on BIO/G-CSF. FN incidence was higher with BIO/G-CSF (61.4%) versus PEG (52.1%) or BIO/PEG (37.5%) (*p* = 0.02 among groups). Patients on BIO/PEG had less frequent grade 2–3 diarrhea (5.5%) compared with BIO/G-CSF (22.5%) or PEG (21.9%); grade 2–3 mucositis was most frequent in the BIO/G-CSF group. In conclusion, pegfilgrastim and its biosimilar displayed an advantageous efficacy and safety profile compared with biosimilar filgrastim in patients with MM post-ASCT.

**Supplementary Information:**

The online version contains supplementary material available at 10.1007/s00277-023-05228-z.

## Introduction

Multiple myeloma (MM) is the main indication for autologous stem cell transplantation (ASCT) worldwide [1, 2], and high-dose melphalan (HDM) (200 mg/m^2^) remains the standard conditioning regimen before transplantation [3–6].

Post-ASCT-related mortality has improved significantly, particularly through new and improved supportive therapies [1, 2, 7]. Use of granulocyte colony-stimulating factors (G-CSF) post-ASCT has been associated with faster neutrophil engraftment in several prospective randomized trials [8, 9], with lower incidences of infection, reduced treatment with broad-spectrum antibiotics, days of hospitalization and treatment costs, and better clinical outcomes [10–13]. Short-acting filgrastim and long-acting pegfilgrastim, among other biosimilars, are approved treatment options for reducing the duration of neutropenia in patients undergoing myeloablative therapy preceding ASCT, who are at an increased risk of prolonged severe neutropenia [14, 15].

Biosimilars are biologic products that are highly similar to an approved originator product with only minor differences in clinically inactive components and no clinically meaningful differences in efficacy, safety, and purity [16]. Since biosimilars are supported by limited clinical data at the time of approval, data must be extrapolated to support their use in additional indications of the originator product. The first biosimilar ever approved was filgrastim-sndz (Sandoz Inc.), a short-acting G-CSF (BIO/G-CSF) that was approved in 2015 [17]. In 2019, the FDA approved pegfilgrastim-bmez (BIO/PEG; Ziextenzo®, Sandoz Inc.), as a biosimilar of the originator product pegfilgrastim (PEG; Neulasta®, Amgen). Biosimilars such as BIO/G-CSF and BIO/PEG are costly, limiting their accessibility for many patients. The increased need for cost-effective hematopoietic growth factors has recently led to the rapid approval of additional biosimilars [18, 19].

Currently, there are no data describing the use of BIO/PEG post-ASCT in patients with MM. This comparative effectiveness study describes the use of BIO/PEG in patients with MM receiving HDM and undergoing ASCT to assess the relative benefits of BIO/PEG in comparison with historic controls (i.e., BIO/G-CSF and PEG).

## Methods

### Patients

This prospective cohort study compared patients receiving BIO/PEG with two groups of historical controls (whose data were collected retrospectively), all of whom were referred to the Stem Cell Transplantation Unit of the Grande Ospedale Metropolitano “Bianchi-Melacrino-Morelli” (GOM-BMM) in Reggio Calabria, Italy, for peripheral blood stem cell collection and ASCT. The study was approved by the local institutional review board and conducted according to the Declaration of Helsinki and the International Conference on Harmonization Guidelines for Good Clinical Practice.

The study included transplantation-eligible patients who were aged 18–70 years, and had de novo MM, who achieved a favorable response after induction therapy (patient response—complete response (CR) or very good partial response (VGPR) and partial response (PR)—was defined according to the International Myeloma Working Group criteria [20, 21]), were stage 1–3 according to the International Staging System, and who had a World Health Organization performance status of 0–2. Patients were excluded if they met any of the following criteria: non-secretory MM; Waldenstrom macroglobulinemia or immunoglobulin M MM; New York Heart Association class II–IV heart failure; abnormal pulmonary function findings; systematic amyloid light-chain amyloidosis; or a history of active malignancy during the past 5 years (excluding basal cell carcinoma or stage 0 cervical cancer). Patients with an absolute neutrophil count (ANC) of ≤ 1.0 × 10^9^/L, a platelet count of ≤ 75 × 10^9^/L, a creatinine clearance of ≤ 15 mL/min, and those with disease refractory to induction chemotherapy were also excluded.

### Treatment

Patients received a bortezomib-based induction therapy; high-dose cyclophosphamide (2–4 g/m^2^) and G-CSF were administered to mobilize peripheral blood stem cells. The minimum target dose of CD34+ cells required to safely support high-dose chemotherapy was 2 × 10^6^/kg. Post-ASCT, patients received a single, 6-mg subcutaneous injection of BIO/PEG, 24 h after stem cell infusion. These patients were compared with two historical control groups consisting of patients who received either BIO/G-CSF 5 μg/kg/day, from day 5 post-ASCT until neutrophil engraftment, or PEG 6 mg, 24 h after ASCT as a single dose. All patients received oral prophylaxis with levofloxacin 500 mg/day from day 0 until neutrophil engraftment and acyclovir 800 mg twice daily from day 3 until approximately day 90 post-ASCT. *Pneumocystis jirovecii* pneumonia prophylaxis was administered with trimethoprim/sulfamethoxazole (1 double strength tablet; 2–3 times weekly), initiated post-hematologic recovery for 3 months. Cryotherapy (ice chips) was utilized to prevent HDM-induced oral mucositis; the patients placed ice chips in their mouths approximately 30 min before and 6 h after HDM. Red blood cell (RBC) and platelet transfusions were administered to maintain hemoglobin levels of ≥ 8 mg/dL and platelet counts of ≥ 10 × 10^9^/L or in patients with symptomatic anemia/minimal mucocutaneous hemorrhagic syndrome. Intravenous hydration and electrolyte support was also provided. Where febrile neutropenia (FN) occurred following a long period of neutropenia (ANC < 0.5 × 10^9^/L or ANC of 1 × 10^9^/L with a predicted decline to < 0.5 × 10^9^/L over the subsequent 48 h) blood and catheter-drawn cultures were ordered, and intravenous ceftriaxone was promptly started.

### Endpoints

The primary endpoint of this study was time to neutrophil engraftment, defined as three consecutive days where the patient had an ANC of ≥ 0.5 × 10^9^/L.

Secondary endpoints included the incidence and duration of FN, and the incidence of mucositis, diarrhea, and platelet engraftment (platelet count ≥ 20 × 10^9^/L, not requiring a platelet transfusion in the preceding 7 days). Complete blood counts were conducted using blood samples collected before chemotherapy and daily during the aplastic phase until hospital discharge. FN was defined as a temperature of ≥ 38.2°C on at least two consecutive occasions, or a persistent temperature of ≥ 38.0°C for at least 1 h, accompanied by an ANC of < 0.5 × 10^9^/L in the absence of any documented noninfectious cause (e.g., transfusion reaction or administration of cytotoxic drugs).

The safety endpoint of the study was the incidence of study drug-related adverse events.

### Statistical analysis

Descriptive statistics were used to present data, including median, interquartile range, and percentage values. Between-group comparisons were performed using the Kruskal–Wallis or chi-square tests, as appropriate. Comparative analyses were used to identify covariates to be included in multiple models as associated with growth factor treatment (BIO/PEG vs PEG; BIO/PEG vs BIO/G-CSF) or FN (yes/no). Accounting for different time frames of drug administration (BIO/PEG was introduced in the last 3 years) and changing treatment guidelines, basal CD34+ infusion was dichotomized based on a clinical threshold over the whole period (< 4 and ≥ 4 × 10^6^/kg).

To assess the relationship between time to neutrophil engraftment and other patient variables, univariate Kaplan–Meier analyses were conducted. As the proportional hazard assumption was violated, the restricted mean survival time (RMST) was adopted to estimate the treatment effect. RMST, defined as the area under the survival function curve up to a specific time (t*), shows the mean survival time or, in our case, the mean time in which there was no neutrophil engraftment. The difference in RMST (∆RMST) can be described as change (gain or loss) in event-free survival time between treatment groups during this specific timeframe. There were no censored observations in this study, so RMST corresponded to the mean survival time and t* corresponded to the total timeframe. To account for differences in follow-up duration between the three treatment groups, a sensitivity analysis was performed over a predefined period (13 days).

The relationship between FN and other patient variables was evaluated by univariate logistic regression analysis; identified covariates were used for multiple logistic regression analysis. For the logistic models, data were expressed as odds ratio (OR), 95% confidence intervals (CI), and *p-*values. All analyses were adjusted by patient sex and age, irrespective of the association with the outcome (significant/not significant).

Statistical analyses were performed with the survRM2 and temporal packages in the software R, version 3.6.3.

## Results

### Study population

From January 2021 to June 2022, 56 consecutive patients with MM underwent HDM followed by ASCT and administration of BIO/PEG. Two historical control groups consisted of 102 patients who underwent transplantation in 2016–2018 and received BIO/G-CSF, and 73 patients who underwent transplantation between 2019 and 2020 and received PEG, both after stem cell infusion.

Table 1 summarizes patient characteristics at the time of ASCT. Patients in all three groups received the same HDM schedule and were adequately matched to historic controls with respect to baseline demographic and clinical characteristics. The proportion of patients with a CR or VGPR and PR was 98.2% (*n* = 55) and 1.8% (*n* = 1) in the BIO/PEG group, 89.2% (*n* = 91) and 10.8% (*n* = 11) in the BIO/G-CSF group, and 95.9% (*n* = 70) and 4.1% (*n* = 3) in the PEG group, respectively.

**Table 1:** Baseline patient characteristics according to treatment

	BIO/PEG	BIO/G-CSF	PEG	*p-*value
	(*n =* 56)	(*n =* 102)	(*n =* 73)	
Males, *n* (%)	32 (57.1)	52 (51.0)	48 (65.8)	0.15
Median age, years (range)	59.5 (53.8–64.5)	60.8 (53.9–63.7)	59.3 (53.9–62.8)	0.70
Myeloma^a^ subtype				
IgG, *n* (%)	36 (64.3)	75 (73.6)	50 (68.5)	0.37
IgA, *n* (%)	12 (21.4)	20 (19.7)	22 (30.1)	
Light chain, *n* (%)	8 (14.3)	7 (6.9)	1 (1.4)	
ISS disease stage^b^				
I, *n* (%)	16 (20.1)	32 (33.0)	28 (43.1)	0.44
II, *n* (%)	29 (52.7)	47 (48.5)	24 (36.9)	
II, *n* (%)	10 (18.2)	18 (18.6)	13 (20.0)	
CD34				
CD34 <4, *n* (%)	14 (25.0)	17 (16.7)	12 (16.4)	0.37
CD34 ≥4, *n* (%)	42 (75.0)	85 (83.3)	61 (83.6)	

*BIO/G-CSF*, biosimilar granulocyte colony-stimulating factor (filgrastim-sndz); *BIO/PEG*, biosimilar pegfilgrastim (pegfilgrastim-bmez); *CR*, complete response; *n*, number; *PEG*, pegfilgrastim; *PR*, partial response; *VGPR*, very good partial response

^a^Not considered for *p-*value calculation as this was the only patient with the light chain myeloma subtype

^b^*p* value was calculated only on valid cases. Percentages reported in the table were calculated on available data (missing value numbers range from 1 to 8)

All patients were in first line of treatment and received 4–6 cycles of a bortezomib induction therapy before mobilization.

### Transfusions

The proportion of patients who underwent RBC or platelet transfusion did not differ between the three treatment groups (Table 2), whereas the median number of platelet transfusions among platelet-transfused patients was twofold higher in PEG-treated patients than in the remaining two groups. There was no difference in the number of RBC transfusions among RBC-transfused patients across the three groups (Table 2).

**Table 2:** Outcome measurements according to treatments^a^

	BIO/PEG	BIO/G-CSF	PEG	*p-*value^b^
	(*n* = 56)	(*n* = 102)	(*n* = 73)	
Hematologic recovery after transplant				
Patients who had RBC transfusions, *n* (%)	10 (18.2)	27 (26.5)	17 (23.3)	0.50
Median (range) RBC transfusions among RBC-transfused patients	1 (1–2)	2 (1–3)	2 (1–3)	0.26
Patients who had PLT transfusions, *n* (%)	23 (41.8)	52 (51.0)	33 (45.2)	0.51
Median (range) PLT transfusions among PTL-transfused patients	1 (1–1)	1 (1–2)	2 (1–2)	< 0.01
Median (range) time to reach platelet count ≥20 × 109/L, days^c^	12 (11–14)	13 (12–14)	13 (11–14)	0.14
Efficacy/safety measurements				
Median (range) time to neutrophil engraftment (ANC ≥ 0.5 × 10^9^/L), days	10 (9–10)	11(11–11)	10 (9–10)	< 0.001
Incidence of febrile neutropenia, *n* (%)	21 (37.5)	62 (61.4)	38 (52.1)	0.02
Median (range) time with fever (≥ 38.2°C), days	2 (1–2)	2 (1–3)	2 (1–3)	0.24
Fever origin, *n* (%)	*n* = 21	*n* = 62	*n* = 38	
Microbiologically/clinically documented	11 (52.4)	10 (16.4)	2 (5.3)^d^	< 0.001
FUO	10 (47.6)	51 (83.6)	36 (94.7)	
Mucositis, *n* (%)				
WHO grade 0	36 (65.5)	9 (8.8)	23 (31.5)	< 0.001
WHO grade 1	15 (27.3)	57 (55.9)	43 (58.9)	
WHO grade 2–3	4 (7.3)	35 (34.3)	7 (9.6)	
WHO grade 4	0	1 (1.0)^e^	0	
Diarrhea, *n* (%)				
WHO grade 0	38 (69.1)	32 (31.4)	22 (30.1)	< 0.001
WHO grade 1	14 (25.5)	47 (46.1)	35 (47.9)	
WHO grade 2–3	3 (5.5)	23 (22.5)	16 (21.9)	

*ANC*, absolute neutrophil count; *BIO/G-CSF*, biosimilar granulocyte colony-stimulating factor (filgrastim-sndz); *BIO/PEG*, biosimilar pegfilgrastim (pegfilgrastim-bmez); *FUO*, fever of unknown origin; *n*, number; *PEG*, pegfilgrastim; *PLT*, platelet; *RBC*, red blood cell; *WHO*, World Health Organization

^a^Percentages reported in the table were calculated on available data

^b^Across groups

^c^Six patients treated with BIO/PEG never had a count below 20 × 10^9^/L

^d^Only two patients treated with PEG had fever (origin documented). PEG was not considered for the single comparison

^e^Not considered for *p-*value calculation as this was the only patient with WHO Grade 4 mucositis

### Time to neutrophil engraftment

**Fig. 1:**
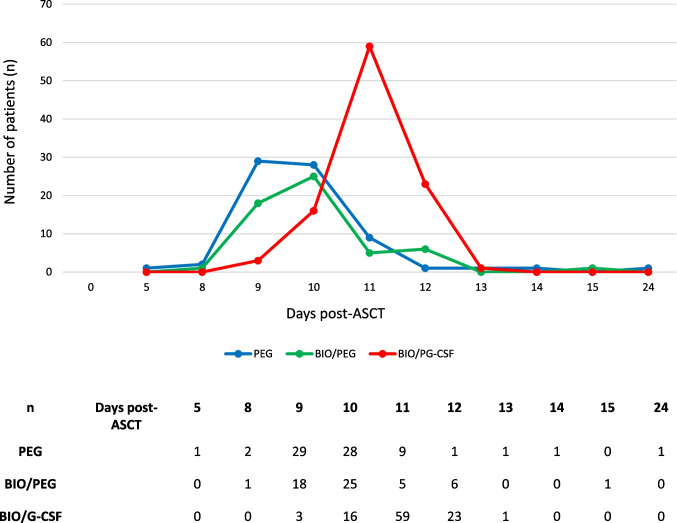
Absolute number of patients reaching neutrophil engraftment (absolute neutrophil count ≥ 0.5 × 10^9^/L) across all three treatment groups over time. *ASCT*, autologous stem cell transplantation; *BIO/G-CSF*, biosimilar granulocyte colony-stimulating factor (filgrastim-sndz); *BIO/PEG*, biosimilar pegfilgrastim (pegfilgrastim-bmez); *n*, number; *PEG*, pegfilgrastim

Figure 1 shows the absolute number of patients achieving neutrophil engraftment (ANC ≥ 0.5 × 10^9^/L) across all three treatment groups over time. In the PEG, BIO/PEG, and BIO/G-CSF groups, the highest proportion of patients achieved neutrophil engraftment at days 9, 10, and 11, respectively. The median time to neutrophil engraftment was 10 days in the BIO/PEG and PEG groups, and 11 days in the BIO/G-CSF group. Among patients who achieved neutrophil engraftment early relative to the median (i.e., day 9; *n =* 50), 29 patients (58.0%) were treated with PEG, 18 (36.0%) with BIO/PEG, and three (6.0%) with BIO/G-CSF (Fig. 1).

Among those who achieved neutrophil engraftment at day 10 (*n =* 69), 28 patients (40.5%) were treated with PEG, 25 patients (36.2%) with BIO/PEG, and 16 patients (23.1%) with BIO/G-CSF. Among those who achieved neutrophil engraftment relatively late (i.e., day 11; *n =* 73), 59 patients (80.8%) were treated with BIO/G-CSF, nine (12.3%) with PEG, and five (6.8%) with BIO/PEG.

**Fig. 2:**
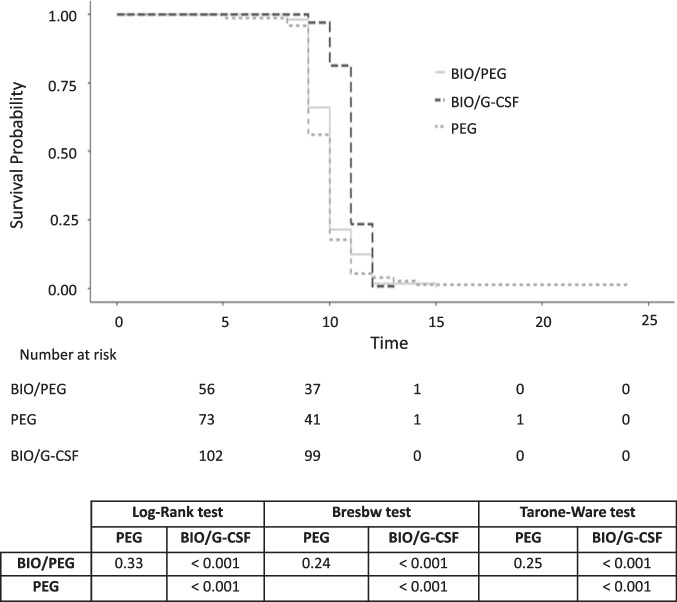
Kaplan–Meier survival analysis of time to neutrophil engraftment (absolute neutrophil count ≥0.5 × 10^9^/L) by treatment group. *BIO/G-CSF*, biosimilar granulocyte colony-stimulating factor (filgrastim-sndz); *BIO/PEG*, biosimilar pegfilgrastim (pegfilgrastim-bmez); *PEG*, pegfilgrastim

The time to reach neutrophil engraftment was further investigated using a Kaplan–Meier analysis (Fig. 2). Cumulative survival-free time of neutrophil engraftment did not differ between patients on BIO/PEG and PEG (log rank test, *p =* 0.33) but was significantly shorter in these groups than in patients treated with BIO/G-CSF (log rank test, *p <* 0.001); i.e., time to neutrophil engraftment was shorter in patients who were treated with BIO/PEG and PEG.

A RMST analysis further confirmed that neutrophil engraftment occurred, on average, 1 day later (*p <* 0.001) in patients treated with BIO/G-CSF than in those treated with BIO/PEG (∆RMST0.99 days; 95% CI 0.66–1.33) and PEG (1.11 days; 95% CI 0.63–1.6); no differences were found between the BIO/PEG and PEG groups (*p =* 0.68; Table 3). Age- and sex-adjusted analyses also confirmed these results.

**Table 3:** Restricted mean survival time estimates by treatment groups

	Time window	RMST (95% CI) (days)	∆RMST (95% CI) (days)	*p-*value
Treatments	*Univariate analyses*			
BIO/G-CSF	15 days	11.03 (10.89–11.17)	0.99 (0.66–1.33)	< 0.001
BIO/PEG		10.04 (9.73–10.34)		
BIO/G-CSF	24 days	11.03 (10.89–11.17)	1.11 (0.63–1.6)	< 0.001
PEG		9.92 (9.46–10.38)		
PEG	24 days	9.92 (9.46–10.38)	−0.12 (−0.67–0.44)	0.68
BIO/PEG		10.04 (9.73–10.34)		
	*Age and sex adjusted analyses*			
Treatments	Time window	Between-arms difference in RMST		
BIO/G-CSF vs BIO/PEG	15 days	1.00 (0.67–1.33)		< 0.001
BIO/G-CSF vs PEG	24 days	1.17 (0.71–1.62)		< 0.001
PEG vs BIO/PEG	24 days	−0.15 (−0.68–0.39)		0.59

*BIO/G-CSF*, biosimilar granulocyte colony-stimulating factor (filgrastim-sndz); *BIO/PEG*, biosimilar pegfilgrastim (pegfilgrastim-bmez); *CI*, confidence interval; *PEG*, pegfilgrastim; *RMST*, restricted mean survival time

To account for differences in time duration between the three treatment groups, a landmark analysis was performed (Supplementary Table S1; Online Resource 1). This sensitivity analysis was carried out over a 13-day period and showed similar results to those reported in the RMST analysis.

### Febrile neutropenia

FN occurred in 52.4% of patients (Table 2). The incidence of FN was higher in patients on BIO/G-CSF (61.4%; 95% CI 52–71%) than those on PEG (52.1%; 95% CI 40–64%) and BIO/PEG (37.5%; 95% CI 25–51%) (*p =* 0.02 among groups). A direct comparison of FN incidence showed that this complication occurred in significantly fewer patients in the BIO/PEG group than in the BIO/G-CSF group (37.5% vs 61.4%, *p <* 0.01). The incidence of FN did not differ significantly between patients in the PEG and BIO/PEG groups (*p =* 0.14) or between those in the PEG and BIO/G-CSF groups (*p =* 0.28).

Among patients with FN, there was no difference in the number of days with fever between the three treatment groups (Table 2). The incidence of fever of unknown origin (FUO) was higher in patients in the PEG group (94.7%) than in the BIO/G-CSF (83.6%) or BIO/PEG (47.6%) groups (Table 2). The incidence of FN did not differ by patient age, sex, or amount of stem cells infused (Table 4).

**Table 4:** Patients characteristics according to febrile neutropenia

	Without FN (95% CI) (*n =* 109)	With FN (95% CI) (*n =* 121)	*p-*value
Males, %	56.9 (47.6–66.2)	57.9 (49.1–66.7)	0.99
Age at transplant, years	59.0 (53.38–62.75)	60.73 (54.77–64.38)	0.12
CD34 ≥ 4, %	78.9 (71.2–86.6)	83.5 (76.9–90.1)	0.47

*CI*, confidence interval; *FN*, febrile neutropenia

As the incidence of FN did not significantly differ between the PEG and BIO/PEG groups, these two groups of patients were combined and compared with the BIO/G-CSF group. In both the univariate and multiple logistic regression analyses, the odds of FN occurring were approximately twofold higher in the BIO/G-CSF group than in the BIO/PEG or PEG groups (Table 5).

**Table 5:** Univariate and multiple logistic regression analysis of febrile neutropenia

		OR	95% CI	*p-*value
Univariate	BIO/G-CSF vs BIO/PEG or PEG	1.89	1.11–3.22	0.02
Multiple (age and sex adjusted)	BIO/G-CSF vs BIO/PEG or PEG	1.86	1.09–3.20	0.02

*BIO/G-CSF*, biosimilar granulocyte colony-stimulating factor (filgrastim-sndz); *BIO/PEG*, biosimilar pegfilgrastim (pegfilgrastim-bmez); *CI*, confidence interval; *OR*, odds ratio; *PEG*, pegfilgrastim

### Mucositis and diarrhea

Significant differences were found in the incidence of mucositis and diarrhea across the three treatment groups (Table 2). Specifically, patients in the BIO/PEG group had a lower incidence of grade 2–3 diarrhea (5.5%) than those in the other two treatment groups (BIO/G-CSF: 22.5%; PEG: 21.9%). The incidence of grade 2–3 mucositis was not significantly different between the BIO/PEG and PEG groups (7.3% vs 9.6%), with a higher incidence in the BIO/G-CSF group (34.3%). No deaths occurred in this study.

### Safety

Mild bone pain was observed in 49.3% (*n =* 36/73) of patients taking BIO/G-CSF and in approximately 10% of patients taking PEG (*n =* 11/102) and BIO/PEG (*n =* 6/56). Bone pain occurred largely on days of neutrophil engraftment. In most patients, pain symptoms were controlled by the administration of paracetamol. No cardiac, neurological, renal, or pulmonary complications were reported, and no patients died in the first 100 days post-transplantation.

## Discussion

In this study, the use of BIO/PEG in patients with MM undergoing ASCT resulted in achievement of neutrophil engraftment (ANC ≥ 0.5 × 10^9^/L) in a median of 10 days, similar to that observed in the historical PEG group. Among patients achieving engraftment earlier, 58.0% were treated with PEG. Furthermore, the use of pegylated G-CSF was associated with a statistically significant faster neutrophil engraftment, as reported in other studies [22, 23].

Current data describing the use of BIO/PEG in patients with MM post-ASCT are scarce. A 2021 study by Wang and colleagues reported that among patients with MM who underwent ASCT, the mean time to neutrophil engraftment was 8.72 days in patients treated with BIO/PEG versus 9.87 days in those who received BIO/G-CSF [24]. Similarly, a comparative study by Vanstraelen and colleagues reported a median time to neutrophil engraftment of 8 and 9 days in patients treated with BIO/PEG and BIO/G-CSF, respectively [25]. The decreased time to neutrophil engraftment in the reported studies may have resulted from variability in a range of factors known to affect engraftment kinetics, including population age, treatment timing, conditioning regimen, stage of disease, and prior melphalan exposure [26, 27]. Combined with our results, albeit in a limited dataset, these data suggest that BIO/PEG has improved efficacy compared with BIO/G-CSF in patients with MM who are undergoing ASCT. In the current study, the cumulative survival-free time until neutrophil engraftment did not differ between patients on BIO/PEG and PEG but was significantly shorter in these groups than among patients treated with BIO/G-CSF. Of note, a pivotal single-dose, three-period crossover study by Bellonand colleagues, which compared BIO/PEG and PEG in healthy adults, found similar pharmacokinetic and pharmacodynamic profiles between the originator and its biosimilar [28]. The equivalent pharmacodynamic profiles observed were indicative of equivalent clinical efficacy, as shown in a confirmatory study of both agents in patients with breast cancer [29]. In the current study, RMST analysis of time to neutrophil engraftment found no significant difference between patients on BIO/PEG and PEG, which is in line with previous studies.

The lowest incidence of FN was in the BIO/PEG group, intermediate in PEG and highest in the BIO/G-CSF, which is also consistent with previously reported clinical data [25, 30, 31]. Although slight differences in patient populations, treatment regimens, and timing/duration of drug administration may result in small variabilities in FN incidence, our data likely reflect the real-world outcomes of patients receiving cytotoxic chemotherapy [32]. The incidence of FN in this study did not differ significantly between patients in the PEG and BIO/PEG groups. Whilst comparative data on these two biosimilars are currently lacking, our observations may in part be supported by the demonstrated pharmacodynamic and pharmacokinetic similarity of the two agents [28].

The incidence of FUO was higher in patients on PEG (94.7%) compared with patients on BIO/G-CSF (83.6%) or BIO/PEG (47.6%). These results differed from those reported by Castagna and colleagues, whereby the FUO incidence was higher with BIO/G-CSF (62%) than with PEG (56%) [33]. This discrepancy between our study and that of Castagna and colleagues may be explained by the latter study including patients with a range of hematologic malignancies and solid tumors, with varying disease stages and differing prior treatments. Further studies into the variables effecting incidence of FUO are warranted.

Patients treated with BIO/PEG had a lower incidence of grade 2–3 diarrhea compared with those on the other two treatments, which is in line with previous observations [29]. The incidence of mucositis was significantly higher in patients treated with BIO/G-CSF than in those treated with BIO/PEG or PEG.

In vivo studies have found that upon administration, recombinant human G-CSF causes an initial transient decrease in peripheral neutrophils [34]. Additionally, peak neutrophil counts typically occur approximately 12 h after BIO/G-CSF administration, after which there is a decline over 2–3 days compared with PEG in which neutrophil counts decline slowly over 1–2 weeks [34]. Further investigation of this hypothesis in prospective, randomized studies is warranted. Finally, bone pain was most commonly observed in patients treated with BIO/G-CSF, consistent with previous reports [35].

Filgrastim is usually administered as a series of daily injections after a chemotherapy cycle, whereas pegfilgrastim (both the original formulation and the biosimilar) is injected once-off, as a single dose, with each chemotherapy cycle. With the convenience of a once-off administration per cycle, BIO/PEG could become the future standard-of-care in an outpatient program for HDM and supportive care after ASCT in patients with MM. This reduced frequency of dosing could result in significant time savings for staff when compared to scheduled daily injections [36–38]. In our study, the pegylated formulation of G-CSF was more effective than BIO/G-CSF as prophylaxis for FN. However, in clinical practice, its usage has been limited because of its higher cost, as the benefit of PEG is unclear compared to its associated cost. Most studies showed that PEG is cost-effective compared with filgrastim as primary and secondary prophylaxis for chemotherapy-induced FN inpatients with lymphoma [39]. Our study did not aim to perform a cost benefit analysis, but it is plausible that there may be an economic advantage in using the pegylated biosimilar formulation in the setting of patients undergoing ASCT [40]. Our findings could assist clinicians and healthcare decision-makers to make informed decisions regarding resource allocation for the management of chemotherapy-induced FN in settings similar to those studied.

Our study has some limitations, including the use of historic control arms. Use of such controls is likely to produce unfair comparisons and confounding variables as we cannot control for differences in patient characteristics between the intervention and control groups. Although control groups were carefully selected to maximize comparability, these results should be extrapolated with caution as the registry data does not account for specific confounders and represents a limited sample size, potentially impacting the efficacy outcomes.

In conclusion, PEG and its biosimilar (BIO/PEG) are advantageous, demonstrating improved efficacy and a superior safety profile compared with BIO/G-CSF in patients with MM who were conditioned with HDM prior to ASCT. Indeed, our work showed a shorter time to reach neutrophil engraftment, a lower incidence of FN, a lower incidence of grade 2–3 diarrhea, and a lower incidence of grade 2–3 mucositis in patients treated with PEG and BIO/PEG. Additional clinical trials, rather than observational studies, would facilitate further acceptance and use of such biosimilars in patients with MM receiving HDM and undergoing ASCT in routine clinical practice.

## Supplementary Information

ESM 1: **Table S1** Restricted mean survival time (RMST) estimates and ΔRMST of univariate and multiple models within 13 days

## Data Availability

The datasets generated during and/or analyzed during the current study are available from the corresponding author on reasonable request.
