# Correction to: Effectiveness of biosimilar pegfilgrastim in patients with multiple myeloma after high‑dose melphalan and autologous stem cell transplantation

**DOI:** 10.1007/s00277-023-05323-1

**Published:** 2023-06-22

**Authors:** Massimo Martino, Mercedes Gori, Gaetana Porto, Maria Pellicano, Ludovica Santoro, Chiara Verduci, Filippo Antonio Canale, Barbara Loteta, Tiziana Moscato, Caterina Alati, Maria Consuelo Ieracitano, Amelia Cuzzocrea, Maria Altomonte, Maria Teresa Florenzano, Antonella Morabito, Giuseppe Irrera, Virginia Naso, Marta Pugliese, Giuseppe Console, Anna Ferreri, Lucrezia Imbalzano, Giovanni Tripepi, Annalisa Pitino

**Affiliations:** 1 Stem Cell Transplantation and Cellular Therapies Unit (CTMO), Department of Hemato‑Oncology and Radiotherapy, Grande Ospedale Metropolitano “Bianchi-Melacrino-Morelli,”, 89124 Reggio Calabria, Italy; 2 Stem Cell Transplant Program CIC587, 89124 Reggio Calabria, Italy; 3 Institute of Clinical Physiology (IFC-CNR), Section of Rome, 00185 Rome, Italy; 4 Hematology Unit, Department of Hemato‑Oncology and Radiotherapy, Grande Ospedale Metropolitano “Bianch I-Melacrino-Morelli,”, 89124 Reggio Calabria, Italy; 5 Pharmacy Unit, Department of Hemato‑Oncology and Radiotherapy, Grande Ospedale Metropolitano “Bianch I-Melacrino-Morelli,”, 89124 Reggio Calabria, Italy; 6 Institute of Clinical Physiology (IFC-CNR), Section of Reggio Calabria, 89124 Reggio Calabria, Italy

**Correction to** **:** **Annals of Hematology**

**https://doi.org/10.1007/s00277-023-05228-z**

The article “Effectiveness of biosimilar pegfilgrastim in patients with multiple myeloma after high‑dose melphalan and autologous stem cell transplantation”, written by Massimo Martino, Mercedes Gori, Gaetana Porto, Maria Pellicano, Ludovica Santoro, Chiara Verduci, Filippo Antonio Canale, Barbara Loteta, Tiziana Moscato, Caterina Alati, Maria Consuelo Ieracitano, Amelia Cuzzocrea, Maria Altomonte, Maria Teresa Florenzano, Antonella Morabito, Giuseppe Irrera, Virginia Naso, Marta Pugliese, Giuseppe Console, Anna Ferreri, Lucrezia Imbalzano, Giovanni Tripepi and Annalisa Pitino, was originally published Online First without Open Access. After publication in volume 102, issue 7, page 1915–1925 the author decided to opt for Open Choice and to make the article an Open Access publication. Therefore, the copyright of the article has been changed to © The Author(s) 2023 and the article is forthwith distributed under the terms of the Creative Commons Attribution 4.0 International License, which permits use, sharing, adaptation, distribution and reproduction in any medium or format, as long as you give appropriate credit to the original author(s) and the source, provide a link to the Creative Commons licence, and indicate if changes were made. The images or other third party material in this article are included in the article’s Creative Commons licence, unless indicated otherwise in a credit line to the material. If material is not included in the article’s Creative Commons licence and your intended use is not permitted by statutory regulation or exceeds the permitted use, you will need to obtain permission directly from the copyright holder. To view a copy of this licence, visit http://creativecommons.org/licenses/by/4.0.

The original article has been corrected.

