# Supplementary material for: Effectiveness of biosimilar pegfilgrastim in patients with multiple myeloma after high-dose melphalan and autologous stem cell transplantation
Source: Ann Hematol. 2023 Apr 20;102(7):1915–25. doi: 10.1007/s00277-023-05228-z (PMC10281896; doi:10.1007/s00277-023-05228-z)
Supplement: Supplementary file 1 — Table S1 Restricted mean survival time (RMST) estimates and ΔRMST of univariate and multiple models within 13 days [file 277_2023_5228_MOESM1_ESM.pdf]

Annals of Hematology

A comparative effectiveness cohort study of biosimilar pegfilgrastim in multiple myeloma patients after high dose melphalan and autologous stem cell transplantation

Electronic Supplementary Material

Authors: Massimo Martino, Mercedes Gori, Gaetana Porto, Maria Pellicano, Chiara Verduci, Filippo Antonio Canale, Barbara Loteta, Tiziana Moscato, Caterina Alati, Maria Consuelo Ieracitano, Amelia Cuzzocrea, Maria Altomonte, Maria Teresa Florenzano, Antonella Morabito, Giuseppe Irrera, Virginia Naso, Marta Pugliese, Giuseppe Console, Anna Ferreri, Lucrezia Imbalzano, Giovanni Tripepi, Annalisa Pitino

Corresponding author: Massimo Martino MD, Stem Cell Transplantation and Cellular Therapies Unit (CTMO) Department of Hemato-Oncology and Radiotherapy, Grande Ospedale Metropolitano "Bianchi-Melacrino-Morelli", Reggio Calabria, Italy. **Email**: massimo.martino@ospedalerc.it

Table S1 Restricted mean survival time (RMST) estimates and ΔRMST of univariate and multiple models within 13 days

		**RMST (95% CI)**	**ΔRMST diff (95% CI)**	***p*-value**
**Treatments**	**Time window**	*Univariate analyses*		
BIO/G-CSF	13 days	11.03 (10.89–11.17)	1.03 (0.72–1.34)	<0.001
BIO/PEG	13 days	10.00 (9.73–10.27)	–	
BIO/G-CSF	13 days	11.03 (10.89–11.17)	1.28 (0.98–1.58)	<0.001
PEG	13 days	9.75 (9.49–10.02)	–	
PEG	13 days	9.75 (9.49–10.02)	-0.25 (-0.63–0.13)	0.2
BIO/PEG	13 days	10.00 (9.73–10.27)	–	
		Age and sex adjusted analyses		
**Treatments**	**Time window**	**Between-group difference in RMST**		
BIO/G-CSFvs.BIO/PEG	13 days	1.04 (0.74–1.34)		<0.001
BIO/G-CSF vs. PEG	13 days	1.31 (1.01–1.62)		<0.001
PEG vs. BIO/PEG	13 days	-0.26 (-0.65–0.12)		0.18

BIO/G-CSF, biosimilar granulocyte colony-stimulating factor (filgrastim-sndz); *BIO/PEG,* biosimilar pegfilgrastim (pegfilgrastim-bmez); *CI*, confidence interval; *diff*, difference; *PEG*, pegfilgrastim; *RMST*, restricted mean survival time
